# Characterization of *cmcp* Gene as a Pathogenicity Factor of *Ceratocystis manginecans*

**DOI:** 10.3389/fmicb.2020.01824

**Published:** 2020-07-31

**Authors:** Zhiping Zhang, Yingbin Li, Laixin Luo, Jianjun Hao, Jianqiang Li

**Affiliations:** ^1^College of Plant Protection/Beijing Key Laboratory of Seed Disease Testing and Control (BKL-SDTC), China Agricultural University, Beijing, China; ^2^School of Food and Agriculture, The University of Maine, Orono, ME, United States

**Keywords:** mango wilt, CRISPR/Cas, cerato-platanin, virulence, hypersensitive response

## Abstract

*Ceratocystis manginecans* causes mango wilt with significant economic losses. In the infection court, cerato-platanin (CP) family proteins (CPPs) are believed to involve in pathogenesis but has not been determined in *C. manginecans*. To confirm this function, a CP protein (CmCP) of *C. manginecans* was characterized in this study. A protoplast of *C. manginecans* was prepared by treating its mycelia with driselase and lysing enzymes. The *cmcp* gene was edited using CRISPR/Cas-U6-1 expression vectors in 60% PEG and 50 μg/mL hygromycin B in the medium, resulting in mutants with *cmcp* deletion (Δ*cmcp*). A complemented mutant (Δ*cmcp*-C) was obtained by transforming *cmcp* to Δ*cmcp*. Both Δ*cmcp* and Δ*cmcp*-C were characterized by comparing them with a wild-type strain on morphology, mycelial growth, conidial production and pathogenicity. Additionally, *cmcp* was transformed and expressed in *Pichia pastoris*, and the derived recombinant protein CmCP caused a severe necrosis on *Nicotiana tabacum* leaves. CmCP-treated plant leaves showed symptoms of hypersensitive response including electrolyte leakage, reactive oxygen species generation and overexpression of defense-related genes *PR-1*, *PAD3*, *ERF1*, *HSR203J*, and *HIN1*. All those results suggested that *cmcp* gene was required for the growth development of *C. manginecans* and functioned as a major pathogenicity factor in mango infection.

## Introduction

*Ceratocystis* spp. attack a wide range of economically important plants, causing cankers, lethal, wilt-type diseases, and black rot of storage roots on many plants. More than 30 plant species of plants representing 14 families are their hosts (Baker et al., 2003; Johnson et al., 2005). *Ceratocystis fimbriata* was firstly reported on mango in Brazil (Viégas, 1960). Subsequently, several other pathogenic species were reported in the world, including *C. manginecans*, *C. acaciivora*, and *C. omanensis* (Tarigan et al., 2011; Van Wyk et al., 2011). Among them, *C. manginecans* is considered as an important pathogen of mango tree and several other plants (Fourie et al., 2016). Mango wilt caused by *C. manginecans* is a serious vascular disease and has caused significantly economic losses to mango industry (Van Wyk et al., 2007; Al Adawi et al., 2013; Zhang et al., 2017).

In the infection course of *Ceratocystis* spp., cerato-platanin (CP) plays a major role, which is a phytotoxic protein secreted by pathogens such as *C. fimbriata* f. sp. *platani*, and induces cell death of tobacco (Pazzagli et al., 1999, 2006). CP is the first member of the cerato-platanin family. Cerato-populin (Pop1) from *C. populicola* is the second type of CP protein secreted by *Ceratocystis* species (Comparini et al., 2009). Up to date, genes codifying for cerato-platanin family proteins (CPPs) have been found in more than 50 fungal genomes (Chen et al., 2013). CPPs are a group of small, secreted, and cysteine-rich proteins. Previous studies have shown that CPPs in fungi are involved in the growth and development of fungi, and the interaction with host plants (Gaderer et al., 2014; Pazzagli et al., 2014). They function as effectors or elicitor molecules found in ascomycetes and basidiomycetes (Wösten, 2001; Pazzagli et al., 2014; Chen et al., 2015). For example, the Epl-1 protein from *Trichoderma harzianum* involves in mycoparasitism, plant resistance induction and self-cell wall protection (Gomes et al., 2015); the HaLP2 protein from *Heterobasidion annosum* induces cell death, autofluorescence and expression of defense genes in host plants (Chen et al., 2015); the elicitor Sm1 from *Trichoderma virens* induces plant defense response and autofluorescence (Djonović et al., 2006); and both SP1 and BcSPl1 induce the production of reactive oxygen species (ROS) and the expression of defense genes of host plants (Wilson et al., 2002; Frías et al., 2011).

CPPs are a well conserved family with a 70% similarity at some conserved motifs (Chen et al., 2013). However, sequence identity is only 13% and sequence similarity is about 40% in the representative members of CP family on protein analysis (Pazzagli et al., 2014). Functions of some genes encoding for CPPs have been confirmed in several fungi, which vary greatly depending on their taxon. For instance, the *mpg1* associates with virulence in *Magnaporthe grisea* (Jeong et al., 2010). The *bcspl1* gene contributes to pathogenicity of *Botrytis cinerea*, and BcSpl1 associates with the plant plasma membrane causing cell shrinkage and chloroplast disorganization (Frías et al., 2011, 2014). On the contrary, the *cu* gene does not directly affect virulence of *Ophiostoma ulmi* (Bowden et al., 1996). The *sp1* gene of *Leptosphaeria maculans* is not crucial for pathogenicity on *Brassica napus* cotyledons (Wilson et al., 2002). In addition, the *mpg1 and cu* genes both associated with “easily wettable” phenotype, which causes by a loss of surface hydrophobicity (Stringer et al., 1991; Bell-Pedersen et al., 1992). Currently, little is known about the function of the gene encoding for CPPs in *C. manginecans.*

As shown in the above studies, gene knock out has been frequently used for studying the function of a gene of interest. Recently, the clustered regularly interspaced short palindromic repeat (CRISPR) system is a promising approach for efficient and precise genome modification (Cong et al., 2013). It has been successfully applied to genome editing in various organisms and cell types, including animals, plants, insects, and bacteria (Carroll, 2014; Cong and Zhang, 2014). This advanced technology has been used on a growing number of filamentous fungi, including several agriculturally important pathogens (Kück and Hoff, 2010; Jiang et al., 2013), such as *Magnaporthe grisea* (Synonyms: *Pyricularia oryzae*) (Arazoe et al., 2015), *Ustilago maydis* (Schuster et al., 2015), and *Alternaria alternata* (Wenderoth et al., 2017). However, less report has been documented in genetic manipulation methods of *Ceratocystis* spp. The purpose of this study was to establish a system of mutation in *C. manginecans* using CRISPR/Cas technology and gene knockout method and characterize the function of *cmcp* gene based on this system.

## Materials and Methods

### Fungal and Plant Materials

*Ceratocystis manginecans* strain MG-1-10 was previously isolated from mango (*Mangifera indica*) and maintained in the Laboratory of Seed Disease Testing and Control (BKL-SDTC), China Agricultural University, and was used as a wild-type parental strain for this study. The fungus was cultured and maintained on malt yeast extract agar (MYEA) (Baker et al., 2003). Tobacco (*Nicotiana tabacum*) plants were maintained at conditions of controlled humidity, temperature, and photoperiod in a growth chamber.

### Characterization of CmCP Protein

The homologous protein of CmCP in *Ceratocystis manginecans* was predicted using BLASTP 2.9 and HMMER 3.1b2 based on sequence similarity and structure similarity (Camacho et al., 2009; Potter et al., 2018). Total RNA was extracted from the mycelium of *C. manginecans* strain MG-1-10 using the Eastep^®^ Super Total RNA Extraction Kit (Promega, Madison, WI, United States). cDNA was reversely transcribed from the RNA using PrimaScript^TM^ RT reagent Kit with gDNA Eraser (Takara, Beijing, China). SIGNALP 4.0 (Petersen et al., 2011) were used for signal peptide prediction. The *cp* orthologs gene (*cmcp*) of strain MG-1-10 and its CDS sequence encoding the mature protein were isolated using the primer pair AM-F and AM-R. A 360-bp fragment from *cmcp*, carrying almost the whole cerato-platanin open reading frame, from the end of the predicted signal peptide to the stop codon, was obtained by PCR from strain MG-1-10 cDNA with primers cp-F and cp-R. Other CPPs sequences were obtained from the GenBank database^1^. Amino acid sequence alignments were generated using ClustalW in MAGA 5.0 (Tamura et al., 2011). Phylogenetic analysis was conducted online using IQ-TREE with the maximum-likelihood method (Nguyen et al., 2015).

### Vector Construction for Gene Replacement

Vectors for replacing the *cmcp* gene were constructed as described by Zheng et al. (2014) and Arazoe et al. (2015) with slight modifications. Briefly, primer pair Cf12-F and Cf12-R was used to amplify a 464-bp region of the *cmcp* gene of genomic DNA of strain MG-1-10. Plasmids PtrpChptA-PItk, pCRISPR/Cas-U6-1 were obtained from other laboratories (Zheng et al., 2014; Arazoe et al., 2015). Deletion vectors for targeting genes were generated as follows. The sequence of 1.7 kb downstream was amplified by thermal asymmetric interlaced polymerase chain reaction (TAIL-PCR) using the Genome Walking Kit (Takara, Beijing, China). Primer pairs M1 + M2 and M3 + M4 were used to amplify a segment of 1.0 kb upstream and 1.0 kb downstream, respectively, of the *cmcp* gene. Similarly, primer pair H1 and H2 was used to amplify a 3.5-kb sequence of *HPH-hsv-tk* DNA from plasmid PtrpChptA-PItk. Additionally, three fragments, including 1.0-upstream, 3.5- *HPH-hsv-tk*, and 1.0-downstream, were combined into a pBluescript SK(−) plasmid using In-Fusion^®^ HD Cloning Kit (Takara, Beijing, China). The recombinant plasmid was cloned into *Escherichia coli* DH5α. With the recombinant plasmid being a template, a 5.5 kb size of the replacement fragment was obtained by PCR amplification using the primer pair M1 and M4. For the pCRISPR/Cas-U6-1 expression vectors of *cmcp* gene, sense and antisense oligonucleotides of target genes were designed using the web-based service CRISPRdirect^2^, and were annealed according to the procedures previously reported (Ran et al., 2013). The annealed oligonucleotides were inserted into plasmid pCRISPR/Cas-U6-1 by Golden Gate cloning, as previously reported (Sakuma et al., 2014; Arazoe et al., 2015). The recombinant plasmid (pCRISPR/Cas-U6-1-SgRNA*cmcp*) was cloned into *E. coli* DH5α. The recombinant plasmid pCRISPR/Cas-U6-1-SgRNA*cmcp* was extracted using the TIANpure Mini Plasmid Kit (TIANGE, Beijing, China). The deletion vectors and pCRISPR/Cas-U6-1-SgRNA*cmcp* expression vectors of the *cmcp* gene were confirmed by PCR and DNA sequencing.

### Protoplast Formation and Transformation

The experiment was conducted following the procedures of Royer et al. (1991) with some modifications. In the optimized procedure of protoplast preparation, eight mycelial plugs (5 mm in diameter) taken from the margin of a 7-day-old colony of *C. manginecans* MG-1-10 were added into a 300-mL flask containing 100 mL complete medium (CM) (Royer et al., 1991). After incubation on a rotary shaker at 100 rpm for 24–30 h at 25°C, mycelia in CM were filtered, washed with 0.5 M MgSO_4_ and treated with enzyme solution, which was prepared by mixing 2% lysing enzymes (Sigma, Beijing, China) and 2.5% driselase (Sigma, Beijing, China) and dissolving them in 0.5 M MgSO_4_. After 3–6 h at 25°C, the enzyme solution was filtered through three layers of Miracloth (Millipore, Beijing, China) to eliminate mycelial residues. The protoplasts in the filtrate were washed with 0.6 M KCl and STC (1 M sorbitol, 25 mM Tris-HCl pH 7.5 and 50 mM CaCl_2_) solutions, and then resuspended in STC at a concentration of 5 × 10^7^ protoplasts/mL.

For an improved method of transformation, DNA fragments of deletion vector (5–10 μg), pCRISPR/Cas-U6-1-SgRNA*cmcp* plasmid mixture (5–10 μg) and heparin (5 μL, 5 mg/mL) were added into 200 μL protoplasts and incubated on ice for 30 min, an aliquot of 2.5-mL PTC (25 mM Tris-HCl pH 7.5, 50 mM CaCl_2_ and 60% PEG 4000) was mixed with the suspension, which was incubated at room temperature for 20 min. The protoplast suspension was diluted with 20 mL STC, and then centrifuged at 4000 rpm for 20 min. The resulting pellet was resuspended in 1 mL OCM [0.5% yeast extract, 0.5% malt extract, 0.132% (NH_4_)_2_SO_4_, 0.6 M sucrose]. After 6 h incubation at 25°C, this transformation mixture was spread on OCMA (OCM plus 1.6% agar) flat plates. After 24–36 h, the plates were overlaid with 12 mL of selective agar (2% malt extract, 0.2% yeast extract, 1% agarose in water containing 50 μg/mL of hygromycin B) and continued to be incubated. Transformants were obtained after 3–7 days post-transformation and transferred to fresh MYEA plates with 50 μg/mL hygromycin B (Supplementary Figure S1). The putative transformants were purified by the single-spore isolation method and confirmed by PCR and quantitative real-time PCR (qRT-PCR) with corresponding primers (Supplementary Table S1). Primer pair M5 and M6 was used to amplify the fragment of the connecting area to confirm that the *HPH-hsv-tk* was inserted into the sites where *cmcp* is normally located in the genome. The results of qRT-PCR were analyzed with the 2^−ΔΔCt^ method using the *18S* rRNA gene as endogenous control (Baccelli et al., 2012).

To confirm that the loss of pathogenesis was due to the deletion of *cmcp* gene, Δ*cmcp* was complemented with a full-length *cmcp* gene (Δ*cmcp*-C). The *cmcp* gene was amplified from the genomic DNA of strain MG-1-10 using primer pair Cf12-F and Cf12-R (Supplementary Table S1). Protoplast transformation of Δ*cmcp* was conducted as described above except that 25 μg/mL F2dU (which supports the growth of the complemented strains Δ*cmcp*-C but not the growth of Δ*cmcp*) was used as a selection agent as previously described (Zheng et al., 2014).

### Fungal Growth and “Easily Wettable” Phenotype

The wild-type *C. manginecans* strain MG-1-10, its *cmcp* deletion mutants (Δ*cmcp*), and the complemented strains (Δ*cmcp-*C) were routinely cultured on MYEA at 25°C for 7 days. To test mycelial growth on MYEA plates, colony diameter was measured after incubation at 25°C for 12 days in an incubator. Each plate was inoculated with a 5-mm-diameter mycelial plug taken from the edge of a 7-day-old colony. There were three replicated plates per treatment, and the colony diameter was perpendicularly measured. Sporulation was counted using a hemocytometer. The procedure of “easily wettable” phenotype was described by Talbot et al. (1993), which was measured in rodA^–^ or Eas^–^ mutants. Three biological replicates were used per experiment.

### Pathogenicity

The wild-type *C. manginecans* strain MG-1-10, Δ*cmcp* and Δ*cmcp-*C were inoculated on healthy mango branches. A small hole was created on those branches using a cork borer, followed by inoculation with a 5-mm-diameter mycelial plug taken from the edge of a 7-day-old culture. There were three biological replicates per treatment. After 10 days of incubation, lesion size was measured. Pathogen-free MYEA plugs were used as a blank control. This experiment was done three times.

### Expression of CmCP in *Pichia pastoris*

The CDS of *cmcp* gene (without signal peptide) was cloned in the *Nco*I and *Xho*I restriction sites of plasmid pPICZ-αAM. The recombinant protein coded by this constructed PCR product contained the *Pichia* α-factor signal sequence at the N-terminus of the *cmcp* sequence and the c-myc and 6 × His epitopes at the C-terminus. The resulting plasmid, pPICZ-αAM-*cmcp*, was linearized with *Sac*I-HF restriction enzyme (NEB, United States) and then transformed into *P. pastoris* X33 strain by electroporation. One of the transformants expressing *cmcp* (X33-pPICZ-αAM-*cmcp*) and one transformant of empty vector or EV (X33-pPICZ-αAM, without *cmcp* insert) confirmed by colony PCR were chosen for all subsequent work.

The supernatant of a culture of the selected transformant, induced for 6 days with 0.5% methanol at 28°C with 250 rpm in BMMY (0.3% K_2_HPO_4_⋅3H_2_O, 1.18% KH_2_PO_4_, 1.34%YNB, 0.4 μg/mL Biotin), was the starting material in the purification of the recombinant protein. CmCP was purified with the aid of the 6 × His tag and carried out with Ni-NTA His∙Bind^®^Resin prepacked column (Millipore, Beijing, China) according to the manufacturer’s instructions. Prior to loading to the column, yeast culture supernatant was adjusted to pH 7.4 by addition of binding buffer (20 mM Na_3_PO_4_, 0.5 M NaCl) and flow through the manufacturer’s instructions at 10 ml min^–1^. After treating with a washing buffer (20 mM Na_3_PO_4_, 0.5 M NaCl, 50 mM iminazole, pH 7.3), the protein was eluted by an elution buffer (20 mM Na_3_PO_4_, 0.5 M NaCl, 500 mM iminazole, pH 7.3), and concentrated by an ultrafiltration tube. The protein solution containing CmCP was confirmed by Western blot (Chen et al., 2015), which was stored at 80°C for later use. Western blot was performed using anti-c-myc antibody produced in rabbit (Sigma, Beijing, China). As a negative control, EV was determined by using the same method as described above. Protein concentrations were determined using BCA Protein Assay Kit (ComWin, Beijing, China), with BSA as a standard. Primers and oligonucleotides used in this study are listed on Supplementary Table S1.

### Infiltration of *N. tabacum* Leaves With CmCP

To study *in vivo* effect of CmCP on *N. tabacum* plant, the protein expressed and purified from X33-pPICZ-αAM-*cmcp* culture medium at concentrations of 60, 12, 2.4, 0.48, and 0.1 μM were infiltrated into *N. tabacum* leaves. The products expressed and purified from EV culture medium and H_2_O were used for controls. The purified protein was infiltrated to a healthy leaf of 5-week-old *N. tabacum* with the aid of a 1-mL syringe without needle. Ten plants per treatment and three biological replicates were used. The treated plants were incubated in a growth chamber under controlled conditions (16-h light/8-h dark period at 22°C with 80% relative humidity).

### Reactive Oxygen Species (ROS) and Electrolyte Leakage

Hypersensitive response (HR) comprises a series of characteristic symptoms that include the induction of ROS and electrolyte leakage (Frías et al., 2011). The generation of H_2_O_2_ and O_2_^–^ was evaluated on CmCP-infiltrated leaves using 3,3′-diaminobenzidine (DAB). CmCP protein solution and the products expressed and purified from EV were infiltrated into *N. tabacum* leaves with a syringe using a half leaf method. Thirty hours later, the leaves were cut and treated with DAB (1 mg/mL, pH 3.8) for 12 h, followed by visualization of DAB deposits. Treated leaves were incubated in ethanol to eliminate chlorophyll prior to photographing. Three biological replicates were employed for each experiment.

To assay electrolyte leakage, *N. tabacum* leaves were infiltrated with CmCP protein solution and the products expressed and purified from EV. After 30 h, 5 leaf disks of 9 mm diameter were cut and submerged into 50 mL water at 28°C on a shaker at 80 rpm. Three hours later, the conductivity was measured with a conductivity meter (Mettleer-Toledo, Shanghai, China). The leaves were treated with boiling water for 20 min, and the conductivity was measured using the same method as described above. Relative conductivity is the ratio of the two results. Three biological replicates were employed in each trial.

### Effect of CmCP on the Expression of Defense-Related Genes in *N. tabacum* Leaves

For the expression of tobacco defense genes upon CmCP or the products expressed and purified from EV infiltration, the infiltrated area of the *N. tabacum* leaves was excised. All the treated leaves were collected at 6, 12, and 24 h post-infiltration (hpi), followed by RNA isolation. cDNA was reversely transcribed from the RNA using PrimaScript^TM^ RT reagent Kit with gDNA Eraser (Takara, Beijing, China). Three biological replicates were performed for each time point and three technical replicates were carried out by qRT-PCR. All qRT-PCR reactions were performed with specific primers (Supplementary Table S1) on an ABI 7500 Fast real-time detection system (Applied Biosystems, Beijing, China). Expression level was analyzed with the 2^−ΔΔCt^ method compared to infiltration with EV-derived protein (set to 1) and using *Tac9* and *EF1*α (Eeongation factor 1 alpha) as reference genes. The induction of five pathogenesis-related genes was studied upon CmCP infiltration: *HSR203J* and *HIN1*, which are considered markers of HR in tobacco (Pontier et al., 1994, 2001), and *PR-1*, which is a pathogenesis-related gene under the control of the transcription coactivator NPR1, the master regulator of systemic acquired resistance (SAR) (Spoel et al., 2009), and *PAD3*, which is a camalexin biosynthesis pathway related gene (Chen et al., 2015), and *ERF1*, which is a marker gene of ET-mediated signaling pathway (Chen et al., 2015).

## Results

### Characterization of CmCP Protein

**FIGURE 1:**
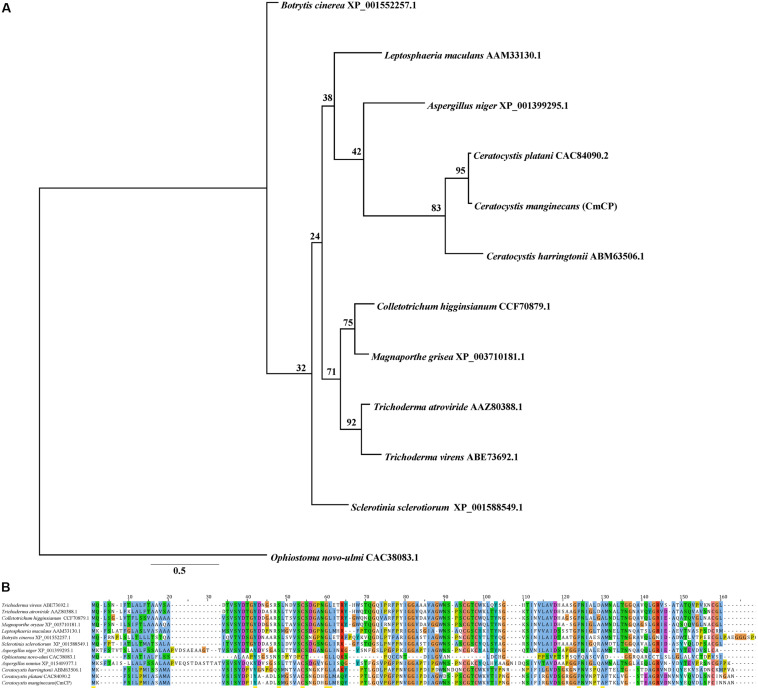
Analysis of the cerato-platanin protein CmCP. **(A)** Evolutionary relationship of CmCP and its homologs from other fungi determined with the maximum-likelihood algorithm. **(B)** Alignment of CmCP and its homologs with ClustalW by MAGA 5.0. Cerato-platanin protein were from: *Trichoderma virens* (GenBank accession number: ABE73692.1), *Trichoderma atroviride* (AAZ80388.1), *Colletotrichum higginsianum* (CCF70879.1), *Magnaporthe oryzae* (XP_003710181.1), *Leptosphaeria maculans* (AAM33130.1), *Botrytis cinerea* (XP_001552257.1), *Sclerotinia sclerotiorum* (XP_001588549.1), *Aspergillus niger* (XP_001399295.1), *Ophiostoma novo-ulmi* (CAC38083.1), *Aspergillus nomius* (XP_015409377.1), *Ceratocystis harringtonii* (ABM63506.1), *Ceratocystis platani* (CAC84090.2).

Bioinformatics analysis showed that there was no homologous protein of CmCP in *C. manginecans*. The CDS sequences of *cmcp* were 405 bp, encoding a protein of 134 amino acids, and contained the cerato-platanin domain. Four cysteine residues putatively involved in disulfide bridge formation were highly conserved. A signal peptide was predicted for CmCP in the first 14 amino acids. Phylogenetic analysis indicated that orthologs of CmCP were widely present in fungi, including some important plant pathogens (Figure 1A). The similarity was 95% between putative mature protein and cerato-platanin (Figure 1B).

### Deletion of *cmcp* Using an Improved CRISPR/Cas System

This was the first report on an improved CRISPR/Cas and homologous recombination system in *C. manginecans*, which generated *cmcp* gene deletion mutants. For the preparation of *Ceratocystis* protoplast, a large number of high-quality protoplasts were obtained from the mycelium treated with enzymes. For the plasmid transfection of *Ceratocystis*, Δ*cmcp* and Δ*cmcp*-C were selected on MYEA plates amended with 50 μg/mL hygromycin B and 25 μg/mL F2dU, respectively.

**FIGURE 2:**
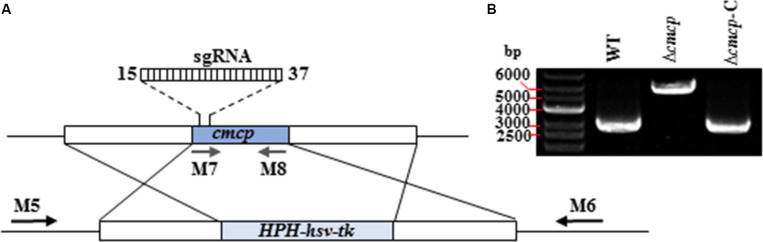
Generation and identification of *Ceratocystis manginecans* mutants with the *cmcp* gene deleted. **(A)** Schematic diagram of replacing *cmcp* gene. The gene replacement cassette *HPH-hsv-tk* contains hygromycin B-resistant gene and herpes simplex virus thymidine kinase gene. Binding sites for primers (M5, M6, M7, and M8) used in polymerase chain reaction (PCR) are indicated by arrows (see Supplementary Table S1 for primer sequences). **(B)** PCR amplicons for Δ*cmcp* transformant and Δ*cmcp-*C strain, using primer pair M5/M6. A 5.9 Kb amplified fragment indicates the *HPH–hsv-tk* was inserted into the sites where *cmcp* is normally located on the genome. WT, wild-type *C. manginecans* strain MG-1-10; Δ*cmcp*, *cmcp*-deletion mutant derived from MG-1-10; Δ*cmcp-*C, *cmcp*-complemented strain of *Δcmcp* mutant.

Deletion vectors and pCRISPR/Cas-U6-1-SgRNA*cmcp* expression vectors of *cmcp* gene were successfully constructed and confirmed by sequencing. Gene-deletion mutants were generated using a CRISPR/Cas system-based homology recombination strategy (Figure 2A). The expected size of the whole transformation fragment was 5500 bp. Using this transformation system (CRISPR/Cas System based homology recombination), 56 hygromycin-resistant transformants were recovered after 3–7 days and genetically purified by single-spore isolation. No colonies were observed on control (no vector DNA fragments) plates. The primers M5 and M6 amplified 5929 bp fragments from the *cmcp* deletion mutants but amplified 2893 bp fragments from the parental wild-type strain MG-1-10 (Figure 2B). The primes M7 and M8 amplified 464 bp fragments on *cmcp* gene of the wild-type strain MG-1-10 but did not amplify any fragments from the *cmcp* deletion mutants. In addition, the same fragments amplified from the wild-type strain MG-1-10 were amplified in *Δcmcp-*C strains (Figure 2B).

In qRT-PCR analyses, the wild-type strain MG-1-10, *Δcmcp* and *Δcmcp-*C were used to quantify the expression of *cmcp* gene using the *18S* gene as endogenous control. Normal expression of *cmcp* gene was detected on the wild-type parental strain MG-1-10 and *Δcmcp-*C strains but no expression was detected of *cmcp* gene in *Δcmcp* mutants. Those results showed that *cmcp* gene were successfully knocked out or complemented in the mutants. The transformation efficiency was higher than 80%.

### Effect of *cmcp* on Mycelial Growth and Conidial Production

**FIGURE 3:**
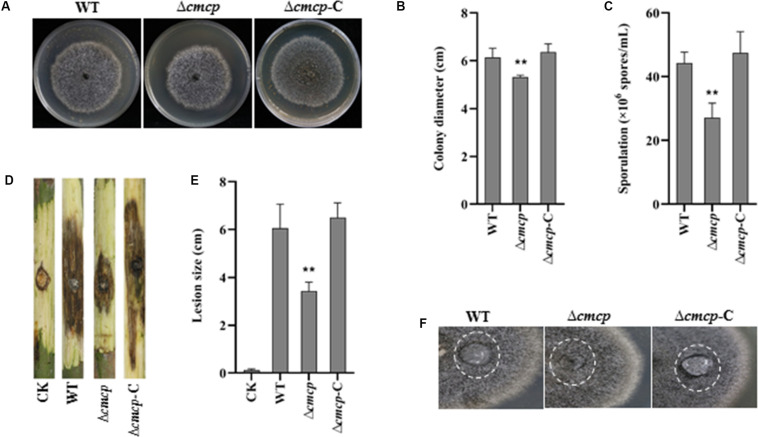
Biological function and virulence of *cmcp* gene in *Ceratocystis manginecans.* Wild-type (WT) *C. manginecans* strain MG-1-10, its *cmcp*-deletion mutant Δ*cmcp*, and *cmcp*-complemented strain Δ*cmcp-*C were incubated on malt yeast extract agar for 12 days at 25°C, resulting in **(A)** culture morphology; **(B)** colony size (*n* = 4 per plot, **P < 0.01, ±SD); **(C)** sporulation (*n* = 4 per plot, **P < 0.01, ±SD); **(D,E)** virulence on mango branches (*n* = 4 per plot, **P < 0.01, ±SD); and **(F)** “easily wettable” phenotype shown in a white circle. Three biological replicates were employed for each experiment.

To examine the effect of *cmcp* on mycelial growth and conidial production, colony diameter was measured, and the number of conidia was counted after incubation at 25°C for 12 days. Δ*cmcp* and MG-1-10 strains reached an average diameter of 5.18 and 6.15 cm, respectively. Δ*cmcp* grew significantly slower than the wild-type strain MG-1-10 on MYEA plates (Figures 3A,B). In addition, conidial production was significantly reduced in Δ*cmcp* compared to MG-1-10 (Figure 3C). After 12 days of incubation on MYEA plates, the number of conidia was 44 × 10^6^ spores/mL and 27 × 10^6^ spores/mL for MG-1-10 and Δ*cmcp* strains, respectively. The *Δcmcp-*C strain showed similar colony diameter and conidial production as the wild-type strain. Therefore, *cmcp* gene might involve in the growth and sporulation of *C. manginecans*.

### “Easily Wettable” Phenotype of *cmcp* Deletion Mutants

To check the hydrophobicity of the Δ*cmcp*, water was placed on the top of fungal cultures. After 12 h, water drop was still suspended on the culture surface of the wild-type strain and *Δcmcp-*C but had soaked into the surface of all the Δ*cmcp* mutants, leaving a pronounced water-soaked mark (Figure 3F). Therefore, the gene product of *cmcp* contributed to cell surface hydrophobicity of the cell surface of mycelium of *C. manginecans*.

### Effect of *cmcp* Gene on the Pathogenicity of *C. manginecans*

The virulence of the wild-type strain and the Δ*cmcp* mutants was studied using mycelial inoculation. After 10 days of incubation, both wild-type strain, *Δcmcp-*C and Δ*cmcp* caused brown or dark lesions on mango branches and many perithecia were observed on the inoculated site. However, virulence of the Δ*cmcp* mutants was significantly reduced (Figures 3D,E). At 10 days post-inoculation, the lesion size caused by wild-type strain and *Δcmcp-*C were 6.05 and 6.5 cm respectively, but Δ*cmcp* produced much significantly smaller lesions on mango branches (*P* < 0.01). No symptom was observed in control branches. Thus, *cmcp* was a major pathogenicity factor in the development of mango wilt.

### Effects of CmCP Treatment on *N. tabacum* Leaves

**FIGURE 4:**
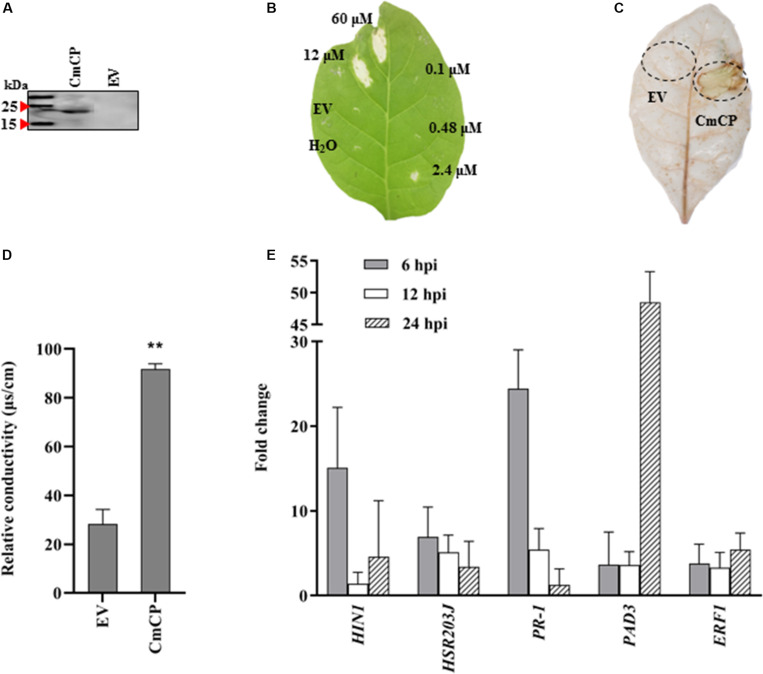
Induction of hypersensitive response (HR) on tobacco (*Nicotiana tabacum*) leaves with CmCP protein. **(A)** Western-blot analysis of the purified recombinant CmCP. **(B)** Tabaco leaves infiltrated with water, EV, and CmCP protein at 0.1, 0.48, 2.4, 12, and 60 μM and incubated for 5 days. **(C)** Reactive oxygen species (ROS) in tabaco leaves infiltrated with 12 μM CmCP or EV for 30 h post inoculation (hpi), detected using 3,3′-diaminobenzidine. **(D)** Electrolyte leakage of tabaco leaves infiltrated with 12 μM CmCP or EV for 30 hpi (*n* = 3 per plot, **P < 0.01, ±SD). **(E)** Expression of defense genes induced by CmCP protein in tabaco leaves detected by quantitative polymerase chain reaction (*n* = 3, **P < 0.01, ±SD). The *y*-axis represents normalized fold (2^−ΔΔCt^) compared to infiltrate with EV (set to 1). *HIN1* and *HSR203J* are markers of HR; *PR-1* is pathogenesis-related gene; *PAD3* is phytoalexin-deficiency gene; and *ERF1* is ethylene response factor 1. EV: the products expressed and purified from empty vector X33-pPICZ-αAM. Three biological replicates were employed for each experiment.

The *cmcp* gene was expressed in *P. pastoris* X33 using the vector pPICZ-αAM-*cmcp*, and the recombinant protein was successfully obtained from yeast culture supernatant (Figure 4A). After being applied to *N. tabacum* leaves, CmCP significantly induced plant cell death at concentrations of 60 and 12 μM but was not effective at 2.4 μM and lower protein concentrations. The necrotic symptoms started to appear at 24 hpi and continued to develop to a larger and visible lesion for several days. A typical symptom at 5 days after infiltration was shown on Figure 4B. Control infiltrations with H_2_O or the products expressed and purified from EV did not produce any lesions on *N. tabacum* leaves. Therefore, cell death of plant leaves was specifically induced by CmCP, and this activity was dose dependent.

### Effects of CmCP Application on ROS and Electrolyte Leakage in *N. tabacum* Leaves

Reactive oxygen species and electrolyte leakage were assayed in plants cells infiltrated with CmCP product. The generation of H_2_O_2_ and O_2_^–^ was evaluated with DAB. DAB staining results showed that an increase in brown DAB precipitate was observed in the area where CmCP had been infiltrated, while control infiltrations with the products expressed and purified from EV did not show any chemical reactions in the leaves (Figure 4C). The production of superoxide anion in tobacco leaves increased at 30 hpi on tobacco leaves with CmCP treatment. Therefore, infiltration of *N. tabacum* leaves with CmCP induced ROS of the treated tissue.

A serious electrolyte leakage was detected at 30 hpi on tobacco leaves with CmCP infiltration. The relative conductivity of CmCP-infiltrated area was significantly higher than the control area infiltrated with the products expressed and purified from EV (Figure 4D). Thus, infiltration of *N. tabacum* leaves with CmCP caused electrolyte leakage of the treated tissue.

### Expression of Defense-Related Genes Induced by CmCP in *N. tabacum* Leaves

The expression of five genes related to plant defenses was examined after *N. tabacum* leaves infiltrated with 12 μM CmCP. qRT-PCR results (Figure 4E) showed that in *N. tabacum* leaves, *PR-1* (pathogenesis-related gene under the control of the transcription coactivator NPR1), *HSR203J* and *HIN1* (marker genes of HR), *PAD3* (phytoalexin biosynthesis pathway) and *ERF1* (Ethylene-mediated signaling pathway) were significantly up-regulated. In the early phase at 6 hpi, transcript levels of *PR-1*, *HIN1* and *HSR203J* reached the maximum at the detecting time point. Relative to reference genes, the transcription of *PR-1* was more than 24 folds above control (the products expressed and purified from EV treated leaves) relative to reference genes; and the transcription of *HIN1* was >15 folds above control. After infiltration for 24 h, *PAD3* expression reached the highest level, and its transcription increased by >40 folds above control. Therefore, CmCP induced the expression of all five genes related to plant defenses.

## Discussion

We have demonstrated that *cmcp* gene was required for growth development of *C. manginecans* and played a key role in pathogenicity of the fungus. The latter was confirmed by the application of CmCP protein that induced necrotic symptoms and HR in *N. tabacum*, including electrolyte leakage, reactive oxygen species generation and overexpression of defense-related genes. We have also demonstrated that an improved CRISPR/Cas system was an effective tool in studying *Ceratocystis*.

Because *Ophiostoma* is a genus genetically close to *Ceratocystis*, we adapted the method of transformation-mediated gene disruption used for *Ophiostoma novo-ulmi*, *cu*^–^ (Royer et al., 1991; Bowden et al., 1996) with improvement. The results were promising. This method showed a high efficiency (80%) in transformation of *Ceratocystis*. Our improvements included (1) using driselase and lysing enzymes instead of NovoZym234 enzyme and 2-mercaptoethanol (strong toxicity) to digest the mycelium (Royer et al., 1991) in protoplast preparation; (2) Because the growth of *C. manginecans* was extremely slow, we used plating the transformation mixture on rather than in the medium (Talbot et al., 1993); and (3) using CRISPR/Cas-U6-1 expression vectors, 60% PEG and 50 μg/mL of hygromycin B (Parsons et al., 1987; Leung et al., 1990; Arazoe et al., 2015). We have shown that using conventional homologous recombination method (not adding pCRISPR/Cas-U6-1-SgRNA*cmcp* expression vectors) and different promoters including 35S and TrpC was unsuccessful. Therefore, we suggested that the improved CRISPR/Cas system we established was an efficient gene editing protocol for *Ceratocystis*.

CPPs play important roles in virulence and mycelial growth and spore formation of fungi (Jeong et al., 2010; Marcos et al., 2011; De O Barsottini et al., 2013; Baccelli, 2015). In this study, we have demonstrated that the virulence of *cmcp* deletion mutants were significantly reduced, as shown in *M. grisea* and *B. cinera* (Jeong et al., 2010; Frías et al., 2011). We also found that *cmcp* deletion mutants possessed a water-soaked phenotype, which was in agreement with others suggesting that CPPs contribute to cell surface hydrophobicity of aerial hyphae of certain plant pathogenic fungi, such as *M. grisea* and *Ophiostoma novo-ulmi* (Talbot et al., 1993; Bowden et al., 1996; Pazzagli et al., 2014). In *M. grisea, mpg1* may involve in attachment, infection court preparation, or topological signaling during infection on plant surface (Mendgen and Deising, 1993; Talbot et al., 1993). In contrast, *cmcp* expression affected mycelial growth and conidial production. Therefore, those phenotypes may be related to the reduced pathogenicity of *cmcp* deletion mutants of *C. manginecans*.

CPPs constitute a well conserved protein family (Chen et al., 2013). However, functions of these genes encoding for CPPs vary greatly depending on the fungal taxon. On the one hand, not all genes encoding CPPs are involved in the pathogenicity of fungi, as confirmed with *sp1* gene in *Leptosphaeria maculans* (Wilson et al., 2002) and *cu* gene in *Ophiostoma novo-ulmi* (Bowden et al., 1996). On the other hand, the growth and overall phenotype are not affected by *epl1* and *epl2* genes in *Trichoderma atroviride* (Frischmann et al., 2013) and *bcsp1* in *B. cinera* (Frías et al., 2011). This may be related to the difference of CPPs in the interaction between pathogen and plant cells, the differences of plant species, or the intrinsic differences in the proteins. *cp* is a single-copy gene in *C. platanin* (Baccelli et al., 2012), which is different with some fungal species containing multiple copies of CPPs homologs (Luti et al., 2020). We have found that there was one predicted *cp* gene in *C. manginecans*. It seems that the number of *cp* gene copy is dependent on fungal species.

By expressing *cmcp* in *P. pastoris*, the function of obtained CmCP product can be examined on *N. tabacum* leaves. Results showed that CmCP-induced necrosis was a dose-dependent activity, and it also induced the expression of defense genes in plant cells. Similar reports have been documented on CPPs from *C. platanin* (Pazzagli et al., 1999), *B. cinerea* (Frías et al., 2011), and *M. grisea* (Yang et al., 2009). CPPs act as microbe/pathogen-associated molecular patterns (MAMPs/PAMPs) (Gaderer et al., 2014; Baccelli, 2015) and induce plant defense responses related to HR, including electrolyte leakage (Frías et al., 2011), reactive oxygen species generation (Djonović et al., 2006) and defenses gene overexpression (Buensanteai et al., 2010; Frías et al., 2011), which supports our results.

We have shown that *HSR203J* and *HIN1* were responsible for HR-induced cell death (Pontier et al., 1994, 2001); *PR-1* is a pathogenesis-related gene associated with salicylic acid-mediated signaling pathways (Spoel et al., 2009); *ERF1* functions via ethylene-mediated signaling pathway (Chen et al., 2015); and *PAD3* is a phytoalexin-related gene (Chen et al., 2015). The expression of all these gene were induced by CmCP. Thus, CmCP may be related to the activation of phytoalexin, and participate in the SA/ET-mediated signaling pathways after infection plant cell. PR1 is a common host protein involved in host defense, and may be a ‘universal’ target to be attacked by pathogen-secreted proteins (Breen et al., 2016; Yang et al., 2017). SsCP1 interacted and targeted plant PR1 and contributes to virulence of *Sclerotinia sclerotiorum* (Yang et al., 2017). Therefore, CmCP can be an effector involved in the process of pathogen-plant interaction, and PR1 should the target of CmCP.

Although CPPs are well-known to act as elicitors or effectors in ascomycete and basidiomycete fungi (Pazzagli et al., 2014), other additional functions should be illustrated. In addition, the low similarity of genes encoding for CPPs leads to the functions of these genes vary greatly in different fungi. Thus, the expression pattern, subcellular localization and structure of CmCP, and its interaction with the host should be further confirmed.

## Data Availability Statement

All datasets generated for this study are included in the article/Supplementary Material.

## Author Contributions

ZZ performed the majority of the experiments, data analysis and preparation of the manuscript. JL provided guidance and designed the experiments. LL, JH, and YL contributed in suggestions in research process as well as in revising the manuscript. All the authors participated in the project proposal and approved the final version of the manuscript.

## Conflict of Interest

The authors declare that the research was conducted in the absence of any commercial or financial relationships that could be construed as a potential conflict of interest.

## Footnotes

1 http://www.ncbi.nlm.nih.gov/

2 http://crispr.dbcls.jp

## Supplementary Material

FIGURE S1 Wild-type *Ceratocystis manginecans* strain MG-1-10 grown on agar plates amended with different concentrations of hygromycin B **(A–L)**: 0, 2.5, 5, 10, 15, 20, 30, 50, 100, 150, 200, and 250 μg/mL.
