# Corrigendum: Characterization of *cmcp* Gene as a Pathogenicity Factor of *Ceratocystis manginecans*

**DOI:** 10.3389/fmicb.2020.595238

**Published:** 2020-09-18

**Authors:** Zhiping Zhang, Yingbin Li, Laixin Luo, Jianjun Hao, Jianqiang Li

**Affiliations:** ^1^College of Plant Protection/Beijing Key Laboratory of Seed Disease Testing and Control (BKL-SDTC), China Agricultural University, Beijing, China; ^2^School of Food and Agriculture, The University of Maine, Orono, ME, United States

**Keywords:** mango wilt, CRISPR/Cas, cerato-platanin, virulence, hypersensitive response

In the original article, there was an error in the Abstract. The word “complemented” was misspelled as “complimented.” A correction has been made to the **Abstract**. The corrected sentence appears below:

“A complemented mutant (Δ*cmcp*-C) was obtained by transforming *cmcp* to Δ*cmcp*.”

The authors apologize for this error and state that this does not change the scientific conclusions of the article in any way. The original article has been updated.

