# Supplementary figures and images for: Characterization of cmcp Gene as a Pathogenicity Factor of Ceratocystis manginecans
Source: Front Microbiol. 2020 Jul 31;11:1824. doi: 10.3389/fmicb.2020.01824 (PMC7411389; doi:10.3389/fmicb.2020.01824)

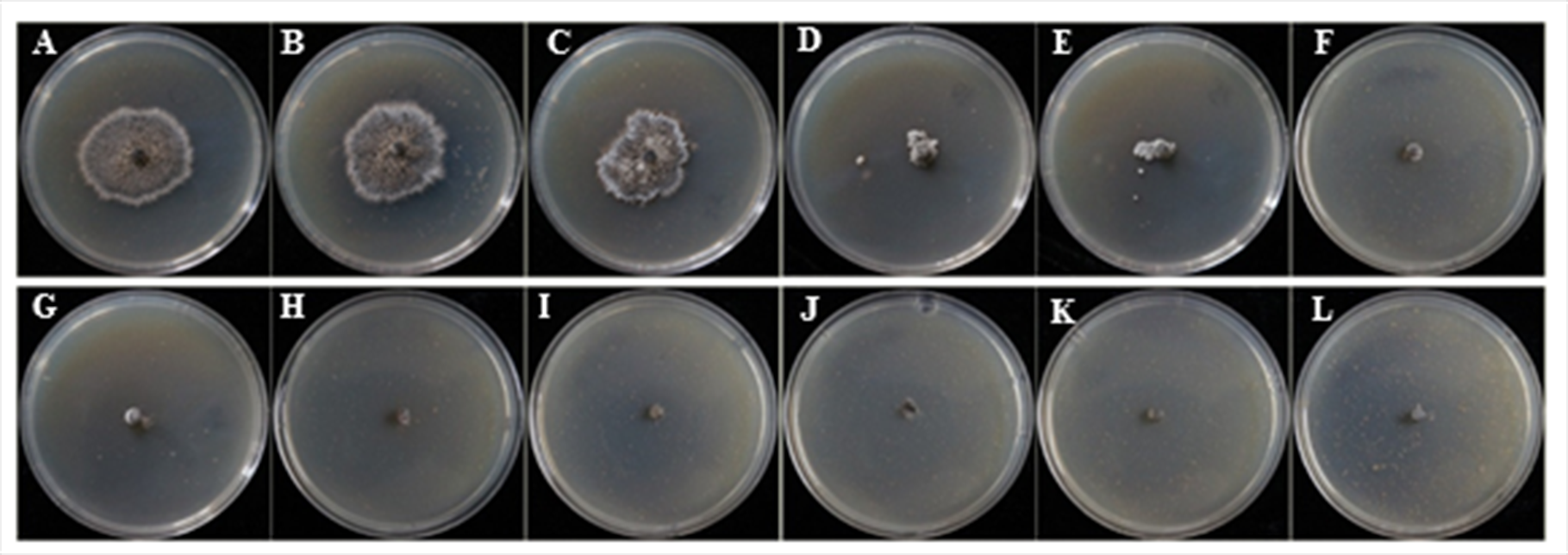

Supplement: FIGURE S1 — Wild-type Ceratocystis manginecans strain MG-1-10 grown on agar plates amended with different concentrations of hygromycin B (A–L): 0, 2.5, 5, 10, 15, 20, 30, 50, 100, 150, 200, and 250 μg/mL. [file Image_1.tif]
