# Supplementary material for: Characterization of cmcp Gene as a Pathogenicity Factor of Ceratocystis manginecans
Source: Front Microbiol. 2020 Jul 31;11:1824. doi: 10.3389/fmicb.2020.01824 (PMC7411389; doi:10.3389/fmicb.2020.01824)
Supplement: Supplementary file 2 [file Table_1.docx]

TABLE S1 | Primers for polymerase chain reaction used in this study

| Primer | Sequence (5'-3') | Target |
| --- | --- | --- |
|  |  |  |
| Cf12-F | CCCAAGCTTATGAAGTTCTCTATCCTA | Full cDNA sequence of *cmcp* |
| Cf12-R | CCGCTCGAGCTAATTAGCGTTGTTAAT |  |
| M1 | CGGTATCGATAAGCTTTTTTGGTCTGTCGACCCTTGAAACA | The upstream *cmcp* fragment |
| M2 | TCTTCTGTTTTGAAAGGTTGGTGGAAGTATGAAAAGAG |  |
| H1 | TTTCAAAACAGAAGATGATATTGAAGGAGCATTTTTTGG | The 3.5-kb *HPH-HSV-tk* fragment |
| H2 | GAAGTCACTGTTCCTTCCGGTATTGTCTCCTTCC |  |
| M3 | AGGAACAGTGACTTCTCCGGAAGCTCCT | Downstream *cmcp* fragment |
| M4 | CGGGCTGCAGGAATTCTATGTTGACTGCCTGCAACAGGC |  |
| M5 | AAGAGGACGAACTCTTACTCTGGTTT | identification of cm*cp* deletion mutants |
| M6 | GTACACATTACCGATTGGTCGCCG |  |
| M7 | ATGAAGTTCTCTATCCTACCCATGATTGCC | *cmcp* gene of *cmcp* deletion mutants |
| M8 | CTAATTAGCGTTGTTAATGCAGAAGGAAAGA |  |
| 18S-F | GGAACAATTGGAGGGCAAGTCT | *18S* expression |
| 18S-R | CAACTACGAGCTTTTTAACCACAACA |  |
| MG-cp-F | GCCAGCGCCATGGCTGTCTCGATCT | *cmcp* expression |
| MG-cp-R | GACCTTCCAGCAAGTACCACA |  |
| AM-F | GGAATTCATGAAGTTCTCTATCCTACC | Full CDS sequence of *cmcp* |
| AM-R | CGCTCGAGATTAGCGTTGTTAATGCA |  |
| cp-F | CCCATGGAAGTCTCGATCTCTTAT | Full CDS sequence of *cmcp* (remove signal peptide) |
| cp-R | GCCTCGAGATTAGCGTTGTTAATG |  |
